# Correction for Ghoshal et al., “9-O-Acetylated Sialoglycoproteins Are Important Immunomodulators in Indian Visceral Leishmaniasis”

**DOI:** 10.1128/msphere.00157-24

**Published:** 2024-05-21

**Authors:** Angana Ghoshal, Sumi Mukhopadhyay, Bibhuti Saha, Chitra Mandal

## AUTHOR CORRECTION

Volume 16, no. 6, p. 889–898, 2009, https://doi.org/10.1128/cvi.00453-08. We are providing this correction to address concerns in Fig. 3 and 4 of this paper that were brought to our attention. Concerns were raised that there is possibly high similarity of a scanty number of dots in the various panels of dot plots showing the flow cytometry analysis in Fig. 3. This is possibly a result of some randomness and noise, as the entire assay and representation are machine generated. The figures are unedited, and the scientific claim of the paper remains unaltered. Possible splicing and duplication concerns were raised for the gel in Fig. 4 representing the mRNA expression data. All the figures are the representative profiles of experiments performed at least three times. The data were presented as such because of the limitations of the equipment (e.g., scanners) and software at the time. Due to the age of the paper and because the lab has been shut down after the retirement of the corresponding author, we do not have access to the original data to rectify the issues within the figures. However, this does not impact the scientific content or the conclusions of this study. We regret any inconvenience this may have caused the readers.

